# Can exercise ameliorate treatment toxicity during the initial phase of testosterone deprivation in prostate cancer patients? Is this more effective than delayed rehabilitation?

**DOI:** 10.1186/1471-2407-12-432

**Published:** 2012-09-26

**Authors:** Robert U Newton, Dennis R Taaffe, Nigel Spry, Prue Cormie, Suzanne K Chambers, Robert A Gardiner, David HK Shum, David Joseph, Daniel A Galvão

**Affiliations:** 1 Edith Cowan University Health and Wellness Institute, Edith Cowan University, 270 Joondalup Drive, Joondalup, Western Australia 6027, Australia; 2 School of Environmental and Life Sciences, The University of Newcastle, Newcastle, NSW, Australia; 3 School of Human Movement Studies, The University of Queensland, Brisbane, QLD, Australia; 4 Department of Radiation Oncology, Sir Charles Gairdner Hospital, Nedlands, WA, Australia; 5 Faculty of Medicine, University of Western Australia, Nedlands, WA, Australia; 6 Behavioural Basis of Health Program, Griffith Health Institute, Griffith University, Brisbane, QLD, Australia; 7 Centre for Clinical Research at Royal Brisbane Hospital, The University of Queensland, Brisbane, QLD, Australia

**Keywords:** Prostate cancer, Androgen deprivation therapy, Exercise, Resistance training, Aerobic training, Side-effects

## Abstract

**Background:**

There has been substantial increase in use of androgen deprivation therapy as adjuvant management of prostate cancer. However, this leads to a range of musculoskeletal toxicities including reduced bone mass and increased skeletal fractures compounded with rapid metabolic alterations, including increased body fat, reduced lean mass, insulin resistance and negative lipoprotein profile, increased incidence of cardiovascular and metabolic morbidity, greater distress and reduced quality of life. Numerous research studies have demonstrated certain exercise prescriptions to be effective at preventing or even reversing these treatment toxicities. However, all interventions to date have been of rehabilitative intent being implemented after a minimum of 3 months since initiation of androgen deprivation, by which time considerable physical and psychological health problems have manifested. The pressing question is whether it is more efficacious to commence exercise therapy at the same time as initiating androgen deprivation, so treatment induced adverse effects can be immediately attenuated or indeed prevented.

**Methods/design:**

We are proposing a multi-site randomized controlled trial with partial crossover to examine the effects of timing of exercise implementation (immediate or delayed) on preserving long-term skeletal health, reversing short- and long-term metabolic and cardiovascular risk factors, and supporting mental health in men receiving androgen deprivation therapy. 124 men who are about to initiate androgen deprivation for prostate cancer will be randomized to immediate or delayed groups. Immediate will commence a 6-month exercise program within 7–10 days of their first dose. Delayed will receive usual care for 6 months and then commence the exercise program for 6 months (partial cross-over). Immediate will be free to adopt the lifestyle of their choosing following the initial 6-month intervention. Measurements for primary and secondary endpoints will take place at baseline, 6 months and 12 months.

**Discussion:**

This project is unique as it explores a fundamental question of when exercise implementation will be of most benefit and addresses both physical and psychological consequences of androgen deprivation initiation. The final outcome may be adjunct treatment which will reduce if not prevent the toxicities of androgen deprivation, ultimately resulting in reduced morbidity and mortality for men with prostate cancer.

**Trial registration:**

ACTRN12612000097842

## Background

There has been a substantial increase in the use of temporary androgen deprivation therapy (ADT) as an adjuvant to radical radiation and surgical therapies for management of prostate cancer [1,2] with substantial periods of ADT now routinely applied to improve outcomes at 5, 10 and 15 years post diagnosis [2-4]. More than 2,000 men in Australia [5] and more than 80,000 in the USA [6] commence on-going ADT for prostate cancer each year. However, ADT leads to a range of well-established musculoskeletal toxicities including reduced bone mass and increased skeletal fractures [7,8] compounded with rapid metabolic alterations including increased body fat, loss of lean mass, insulin resistance and negative lipoprotein profile [9-15]. Recent work, including our own current Australian cohort, suggests an increased incidence of cardiovascular and metabolic morbidity associated with temporary ADT [16-20] and we have also reported significantly increased distress [21] and reduced quality of life (QOL) [22,23]. Our team has shown that even a 9-month exposure to ADT leads to significant reductions in bone mass across different clinical sites concurrent with severe loss of lean mass and increased trunk and whole body fat mass, all surrogate indicators of osteoporosis/skeletal fractures and cardiovascular/metabolic complications [15]. Currently, there is no established treatment to reverse bone loss and the array of metabolic adverse effects associated with severe hypogonadism from temporary ADT. Preliminary clinical trials by our team [24-26] and others [27,28] have suggested high efficacy of exercise for these patients but evidence is limited to only a few studies with men on long-term androgen deprivation [24,27-29]. We have shown that a combined program of resistance and aerobic exercise leads to a number of significant and clinically meaningful benefits including reversal of muscle loss in men receiving ADT for an average of approximately 14 months [24]. A critical but as yet unanswered research question is to determine whether it is more efficacious to commence exercise therapy at the onset of androgen deprivation so treatment induced adverse effects can immediately be attenuated or even completely prevented. This has not been addressed in any exercise trials to date and has the potential to prevent much of the ADT toxicities from the outset rather than try to rehabilitate the patient from the effects of long-term ADT later. A recent report [30] showed that physical function and quality of life are compromised within 3 months of commencing ADT suggesting that up-front exercise interventions are needed to counteract these losses, as well as the marked reductions in bone density and bone strength. Importantly, it appears the initial beneficial effects of resistance and aerobic exercise programs are similar for neuromuscular and physical function regardless of whether patients are on acute (3–6 months) or chronic (>6 months) ADT [31]. Such preliminary evidence supports the hypothesis that exercise might be best initiated when ADT commences, to enhance physical function, retain structure and improve the patient’s acceptance of hormone therapy. This is an important finding as it suggests that exercise may still benefit men during acute ADT, but no research has trialled this from time zero; that is initiation of ADT. This is a considerable gap in our understanding of the management of prostate cancer and ADT.

Having successfully completed several pilot studies, a randomised controlled trial (RCT) and ongoing RCTs in prostate cancer [24-26,31-34], this trial will drill down to the specifics of exercise as medicine to improve skeletal health, physical function, quality of life and mental health implemented immediately when patients initiate ADT. We propose a RCT with partial crossover to examine the effects of the timing of exercise implementation. We will evaluate the following hypotheses:

1) It is more efficacious to commence exercise therapy at the onset rather than after six months of ADT; and

2) ADT side effects, in particular the substantial initial bone loss, can be prevented by a 6-month exercise program concurrently undertaken at the onset of ADT.

The primary endpoint will be spine and hip aBMD determined by DXA. Secondary endpoints will include: 1) volumetric BMD (vBMD) and micro-architecture at the tibia, 2) body composition (lean mass and fat mass/abdominal obesity), 3) blood pathology (glucose metabolism, lipid profile, prostate specific antigen (PSA), testosterone, bone formation and resorption markers), 4) physical function, muscle strength and balance, 5) physical activity level and motivation, and 6) health-related quality of life and psychological distress.

The ultimate outcome will be guidelines for the prescription of exercise for the prevention of ADT toxicities, primarily those related to long-term skeletal health and physical function. This project is unique as it explores a fundamental question of when exercise implementation will be of most benefit to men undertaking ADT. The final outcome may be an adjunct treatment which will prevent major toxicities of ADT, ultimately resulting in reduced morbidity and mortality for men with prostate cancer.

## Methods/design

We are proposing a single-blinded (investigators and testing personnel blinded to group allocation) RCT with partial crossover to examine the effects of the timing of exercise implementation. An immediate exercise group (IE) will undertake the exercise program for 6 months. After 6 months, the delayed exercise group (DE) will be crossed to receive the same intervention program (Table 1).

**Table 1:** Summary of the Intervention Arms

	**0**	**6**	**12**
**Immediate Exercise (n=62)**	Exercise Intervention		No formal intervention
**Delayed Exercise (n=62)**	Usual Care		Exercise Intervention

### Recruitment

**Figure 1:**
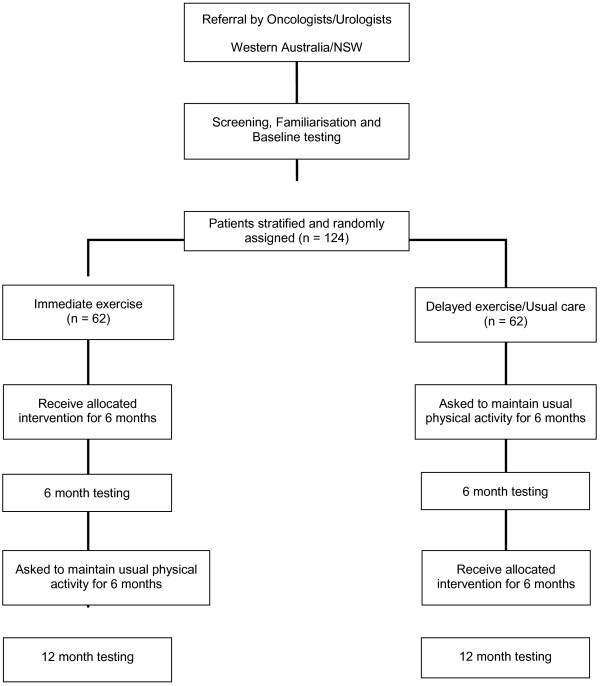
CONSORT Diagram.

Subjects will be recruited by invitation of their specialist (radiation oncologist/urologist) as previously reported in completed and ongoing trials [24,26,32]. Those entering the study will undertake a series of familiarisation sessions and baseline measurements prior to randomisation (Figure 1).

### Randomisation and stratification

Patients will be randomly allocated in a ratio of 1:1 to the two treatment arms for IE and DE groups, subject to maintaining approximate balance regarding stratification for age (<=70 yr>) and smoking status (yes/no). A research methods consultant will be responsible for this randomisation and confirming the groups are balanced on these parameters. The chief investigators, exercise physiologists, psychologists and other researchers conducting the study measures will be blinded to a given participant’s group allocation. The exercise intervention will be provided by exercise physiologists not in the research team or performing the tests (single blinded).

### Subjects

One hundred and twenty-four men (62 subjects per arm) beginning treatment for prostate cancer involving ADT with no regular exercise (undertaking structured aerobic or resistance training two or more times per week) within the past 3 months will be recruited by invitation of their attending specialist in the Perth, Western Australia, and the Central Coast region of New South Wales. All participants will be able to walk 400-m and will require physician consent. Exclusion criteria will include prior exposure to ADT (e.g. those re-initiating ADT from intermittent programs), existing hypogonadism, established metastatic bone disease, established osteoporosis, those taking medications known to affect bone metabolism (e.g. bisphosphonates), acute illness or any musculoskeletal, cardiovascular or neurological disorder that could inhibit or put them at risk from exercising. The protocol has been approved (ID: 7869 NEWTON) by the University Human Research Ethics Committee and all subjects will provide written informed consent.

### Measurements

All measurements for primary and secondary endpoints will take place at baseline, 6 and 12 months. Additional blood measures, scans and questionnaires will also be undertaken at 3 months to track changes over time during the initial 6 months for bone formation and resorption markers. Body composition measures for muscle and fat mass will also be performed at 3-month intervals to track the trajectory of change in these outcomes.

### Calcium and vitamin D

All participants will receive standard daily supplementation with calcium (1,000 mg/d) and vitamin D_3_ (800 IU/d).

### Primary study endpoints

#### Areal BMD

BMD (g/cm^2^) of the hip (total hip) and lumbar spine (L_2-4_) as well as whole body bone mineral content (BMC, g) will be assessed by DXA (Hologic Discovery A, Waltham, MA). The Instant Vertebral Assessment (IVA) and Quantitative Morphometry (QM) program will be used to determine the presence or absence of vertebral fractures prior to initiation of the study.

### Secondary study endpoints

#### Volumetric BMD and bone architecture

Three dimensional pQCT (XCT3000, Stratec, Pforzheim, Germany) will be used to measure volumetric BMD and micro-architecture at the tibia. This technique provides additional data on trabecular and cortical density and geometry with actual prediction of fracture thresholds. Methods for analysis will be as previously described [35].

#### Body composition

In addition to BMD, regional and whole body lean mass (including appendicular skeletal muscle mass) and fat mass will be derived from the whole body DXA scan. Measurement of trunk adiposity is an important indicator of chronic disease risk, and will be assessed from trunk fat mass obtained from the whole body scan and the ratios of trunk fat to limb fat, and trunk fat to total fat. Images from the pQCT scans will also be analysed for muscle density and cross-sectional area for the lower limbs.

#### Blood markers

Testosterone, prostate specific antigen (PSA), insulin, glucose, haemoglobin A1c (HbA1c), C-reactive protein (CRP), bone formation [alkaline phosphatase, Pro collagen Type 1 N-Terminal Pro peptide (PINP)] and resorption [C-terminal telopeptide of type I collagen (CTX)] markers, vitamin D and lipid profile levels will be measured commercially by an accredited Australian National Association of Testing Authorities (NATA) laboratory (Pathwest Diagnostics, Perth, Western Australia) [24].

#### Blood pressure and arterial stiffness

A validated oscillometric device (HEM-705CP, Omron Corporation, Japan) will be used to record brachial BP at the dominant arm in triplicate. Central (ascending aortic) BP and indices of arterial stiffness will be determined using a SphygmoCor system (AtCor Medical, Sydney, Australia). Radial artery pressure waveforms will be captured at the right arm by applanation tonometry using a high fidelity micromanometer (SPC-301, Millar Instruments, Houston, Texas, USA). A generalised transfer function is applied to the radial artery waveform in order to obtain the pressure waveform at the ascending aorta. This method has been validated against invasive techniques for determination of central BP and the augmentation index (AIx) is a marker of systemic arterial stiffness.

#### Muscle strength and balance

Prior to muscle testing, subjects will be familiarised with all assessment procedures. In addition, a warm-up consisting of aerobic activity and stretching will be undertaken. Dynamic concentric muscle strength for the leg press, chest press and seated row undertaken in the program will be measured using the one repetition maximum (1-RM) method. The 1-RM is the maximal weight an individual can move through a full range of motion without change in body position other than that dictated by the specific exercise motion [36]. A Neurocom Smart Balancemaster (Neurocom, OR, USA) will be used to assess standing balance. This device measures ground reaction force to track whole body centre of pressure and a tilting visual field and support platform to separate the visual, somatosensory and vestibular balance sense of the patient. Falls self-efficacy will be determined using the Activities-Specific Balance Confidence scale. During the course of the intervention, all participants will record any falls that take place and submit monthly fall records to the investigators.

#### Objective measures of physical function

A battery of tests will be used to assess functional performance [24,37]. Tests will be performed in triplicate (except for the 400-m walk which will be performed once) with sufficient recovery time between trials. The best performance on each test will be used in the analyses. The tests will be; 1) repeated chair rise, 2) stair climb, 3) 6-m backward tandem walk, 4) 6-m walk, usual and fast pace, and 5) 400-m walk. Performance in each test will be timed electronically using a Kinematic Measurement System (Fitness Technology, Australia)

#### Physical activity level and motivation

Self-reported physical activity will be assessed by the leisure score index from the Godin Leisure-Time Exercise Questionnaire. ActiGraph activity monitors (triaxial accelerometer, GT3X+, Actigraph, Pensacola, Florida) will be used to objectively assess physical activity levels and sedentary time over a 7-day period [38]. A 6-item questionnaire will be used to assess the domain-specific sedentary behaviour.

The Theory of Planned Behaviour (TPB) is the most widely utilised behavioural framework when examining physical activity motivation in cancer survivors. Therefore, physical activity motivation will be assessed in accordance with the TPB. TPB constructs (affective and instrumental attitude, injunctive and descriptive norm, self-efficacy, perceived behavioural control, intention, and planning) will be assessed in accordance with established guidelines using standardised items [39].

#### Health-related quality of life and psychological distress

Health-related QOL will be measured using the Medical Outcomes Study Short-Form 36 (SF-36), European Organisation for Research and Treatment of Cancer (EORTC) QLQ-C30 and EORTC QLQ-PR25 as well as a health history questionnaire. This validated instrument is an integrated system to assess QOL in cancer patients and has been extensively employed in clinical trials [40]. The Brief Symptom Inventory-18 (BSI-18) will be used to assess psychological distress (Anxiety, Depression and Somatisation) [21]. Higher somatization and anxiety as measured by the BSI-18 is associated with greater physical inactivity in cancer survivors [21]. The Impact of Events Scale (IES) and the Memorial Anxiety Scale for Prostate Cancer (MAX-PC) will be used to measure cancer specific distress [41,42]. A core self-evaluation questionnaire and lifestyle individual resilience scale will be used to assess core self-evaluation and three related aspects of resilience (individual, social–peers and social–family resources). Fatigue will be assessed using the Functional Assessment of Chronic Illness Therapy-Fatigue (FACIT-F) questionnaire. The FACIT-F is a 13 item scale commonly used to assess fatigue in cancer patients [43] as well as cancer patients receiving exercise interventions [44]. Items from the Pittsburgh Sleep Quality Index (PSQI) will be used to measure sleep quality [45]. The PSQI is used to assess quality of sleep over a 1-month interval, and has been shown to be reliable and sensitive to change [46].

### Exercise intervention

The intervention program will comprise resistance, aerobic and impact-loading exercises undertaken 3 times per week in an exercise clinic. The frequency of resistance and aerobic exercises will alternate weekly so two aerobic/impact loading and one resistance/impact loading sessions are performed in the first week and two resistance/impact loading and one aerobic/impact loading are performed in the subsequent week. Resistance training sessions will take approximately 60 minutes (this includes the warm-up and cool-down periods) and will be conducted in the Exercise Clinics at Edith Cowan University (ECU) in Perth and seven other partner sites in Western Australia: Perth; Mandurah; and Bunbury. We also have another site at the University of Newcastle where we have identical equipment and procedures. The programme will include exercises such as leg press, leg extension, leg curl, chest press, seated row, lat pulldown and bicep curl that target the major upper and lower body muscle groups, which we have used in a number of previous studies [24,26,37,47] including men on ADT. To ensure the progressive nature of the training program, subjects will be encouraged to work past the specific repetition maximums (RMs) prescribed. The resistance will be increased by 5-10% increment for the next set/training session if the subject is able to perform more repetitions than the RMs specified during a set. Intensity will be manipulated from 6-12-RM (e.g. the maximal weight that can be lifted 6 to 12 times) using 1–4 sets per exercise. The aerobic component will include 25–40 minutes of cardiovascular exercise using various modes such as walking or jogging on a treadmill, cycling or rowing a stationary ergometer, or exercising on a cross training machine. Target intensity will be 60-85% estimated maximum heart (220–age) with individual heart rate watches (Polar Electra Oy, Finland) provided for each participant. In addition to the clinic training, participants will be encouraged to undertake twice weekly home-based training incorporating aerobic activity (e.g. walking, cycling) and a modified version of the impact-loading regimen (only including hopping, leaping and drop jumping) for the duration of the study. The impact-loading regimen will be performed a minimum of 3 times per week for the duration of the trial, in combination with the resistance and aerobic exercise. For the first 10 weeks, 2 rotations will be performed of skipping (30 sec), bounding over soft hurdles (13–16 cm), and drop jumping (10–15 cm). In the second 10-week period, hopping on one leg (10 times) will be added, leaping (10 times) will replace skipping, and 4 rotations of bounding (19–25 cm), drop jumping (20–25 cm), hopping, and leaping will be performed for the remainder of the programme. These activities result in substantial peak ground reaction forces ranging from 3.4 to 5.2 times body weight providing excellent stimulus to bone yet proven safe and accepted by older people [48].

All exercise sessions will be conducted in small groups of up to 6–10, with participants exercising in pairs and under direct supervision to ensure correct technique and minimize the risk for injury. Each session will commence with a 10-minute warm-up comprising low-level aerobic activities such as walking and stationary cycling, as well as stretching and conclude with a 5-minute cool-down period of stretching activities. In order to reduce the possibility of boredom and overreaching the exercise program will be periodised by cycling emphasis on intensity and volume. Also, within sessions variations of circuit training and intermittent exercise sessions (intervals of high and low intensity exercise) will be introduced. The exercise program will be designed to provide optimal stimulus to the skeletal, cardiorespiratory and neuromuscular systems while maximizing compliance and retention. All participants will be asked to maintain customary physical activity and dietary patterns over the intervention period (apart from the programmed exercise). Physical activity and dietary intake will be assessed at baseline, 6 and 12 months. During the course of the study, participants will be required to maintain an activity log and record their recreational physical activities. Participants in the DE group will be contacted every 4 weeks to encourage them to maintain current physical activity levels and record their activities. Dietary intake, at the same time points as for physical activity, will be assessed using a 4-day dietary record. Dietary information will be derived using the FoodWorks software program.

### Calculation of sample size

Data from our 36-week study [15] in prostate cancer survivors initiating ADT indicates that the standard deviation (SD) for change in our primary outcome of BMD equates to approximately 4.5% and 3.3%, for the hip and lumbar spine, respectively. With ADT, the annual loss is reported to be 2-8% at the spine and 1.8-6.5% at the hip [49,50] and based on our 36-week data, we obtained an initial loss of 1.5% and 3.9% for the hip and lumbar spine, respectively. Therefore, we anticipate losses of approximately 1.5% and 3.5% at the clinically relevant fracture sites of the hip and spine. We anticipate a difference between the immediate and delayed exercise groups of approximately 2.5% at the hip and 4.5% at the lumbar spine, which would be clinically significant and substantially reduce the risk for fracture. A priori, 51 subjects per group will be required to achieve 80% power at an alpha level of 0.05 (two-tailed), and to demonstrate a difference between groups at each bone site at the end of the 6-month intervention. Previous experience in our exercise trials indicates an attrition rate of up to 20% over the course of the study period. Therefore, to adequately ensure that we have sufficient subject numbers at the end of the intervention, 124 subjects will be randomised in a ratio of 1:1 to the immediate and delayed exercise groups, respectively. A sample size of 124 will also provide us with sufficient power to detect differences in our secondary outcomes which all have larger effect sizes based on our previous research.

### Statistical analysis

Data will be analysed using SPSS statistical software package and an intention-to-treat approach will be applied. Analyses will include standard descriptive statistics, Student’s *t* tests, correlation and regression, and two-way (group x time) repeated measures ANOVA (or ANCOVA as appropriate) to examine differences between groups over time. All tests will be two-tailed and an alpha level of 0.05 will be applied as the criterion for statistical significance.

## Discussion

This is the first intervention using a combination of resistance, aerobic and impact loading exercise implemented immediately with initiation of ADT as opposed to long-term androgen deprivation. The principal outcome of this project will be the determination of whether it is more efficacious to commence exercise therapy at the onset of ADT so treatment induced adverse effects can be immediately attenuated or even completely prevented. This is an exciting possibility. Second, this is the first study to our knowledge using pQCT to assess bone outcomes of a therapeutic exercise intervention in a cancer population. Importantly, this simple and cost effective intervention strategy may provide comparable benefits to pharmaceutical interventions (e.g. bisphosphonates) without exposing patients to additional potential side effects [36,51,52] or the high financial cost of these drugs. The most important outcome will be clinical guidelines for the concurrent prescription of exercise for the management of men initiating ADT to preserve long-term skeletal health, reduce metabolic and cardiovascular morbidities, maintain physical function and alleviate psychological distress and depression associated with severe hypogonadism resulting from temporary ADT. By examining psychological outcomes of depression and distress we are addressing all aspects of ADT toxicities in the initiation phase, an important time when patient discomfort is greatest but unfortunately not addressed to date. This holistic approach to ADT toxicity will result in more effective clinical guidelines for managing patients, in particular maximizing uptake and long term adherence of exercise therapy. In terms of advancement of prostate cancer care, we expect dissemination of the knowledge gained from this project to reduce fracture risk, improve physical and functional ability, quality of life, mental health and ultimately survival rates in this population. In particular, we hope to establish that exercise implemented as men initiate ADT can offer an array of positive patient outcomes and this strategy is far superior to the current delayed rehabilitation approach. Such benefits, apart from enhancing quality of life, could significantly reduce health care costs and ultimately increase survivorship.

## Abbreviations

ADT: Androgen deprivation therapy; QOL: Quality of Life; RCT: Randomized Controlled Trial; IE: Immediate Exercise Group; DE: Delayed Exercise Group; PSA: Prostate Specific Antigen; DXA: Dual Energy X-ray Absorptiometry; ABMD: Areal Bone Mineral Density; pQCT: Quantitative Computed Tomography; vBMD: Volumetric Bone Mineral Density; IVA: Instant Vertebral Assessment (IVA); QM: Quantitative Morphometry (QM); Hba1c: Hemoglobin A1c; CRP: C- reactive protein; PINP: Pro Collagen Type 1 N-Terminal Pro Peptide; CTX: C-terminal Telopeptide of Type I Collagen; 1-RM: One Repetition Maximum; IES: Impact of Events Scale; MAX-PC: Memorial Anxiety Scale for Prostate Cancer; SF-36: Medical Outcomes Study Short-Form 36; EORTC: European Organisation for Research and Treatment of Cancer; FACIT-F: Functional Assessment of Chronic Illness Therapy-Fatigue; TPB: Theory of Planned Behaviour; BSI-18: The Brief Symptom Inventory-18; LL-FI: The Late Life - Function Index; PSQI: Pittsburgh Sleep Quality Index.

## Competing interests

The author(s) declare that they have no competing interests.

## Authors’ contributions

RUN, DRT, NS and DAG developed the study concept and protocols and initiated the project. DJ, RAG, DHKS, PC and SKC assisted in further development of the protocol. RUN, DRT, NS, PC and DAG drafted the manuscript. NS, DJ, SKC, DHKS and RAG will provide access to patients. RUN, DRT, PC and DAG will implement the protocol and oversee collection of the data. All authors contributed to and approved the final manuscript.

## Pre-publication history

The pre-publication history for this paper can be accessed here:

http://www.biomedcentral.com/1471-2407/12/432/prepub
